# Natural aging and Alzheimer’s disease pathology increase susceptibility to focused ultrasound-induced blood–brain barrier opening

**DOI:** 10.1038/s41598-023-30466-6

**Published:** 2023-04-25

**Authors:** R. L. Noel, A. J. Batts, R. Ji, A. N. Pouliopoulos, S. Bae, A. R. Kline-Schoder, E. E. Konofagou

**Affiliations:** 1 Department of Biological Engineering, Columbia University, 351 Engineering Terrace, Mail Code 8904, 1210 Amsterdam Avenue, New York, NY 10027 USA; 2 Department of Radiology, Columbia University, 622 West 168th Street, New York, NY 10032 USA

**Keywords:** Biomedical engineering, Neuroscience, Alzheimer's disease

## Abstract

Focused Ultrasound (FUS) paired with systemically-injected microbubbles (μB) is capable of transiently opening the blood–brain barrier (BBBO) for noninvasive and targeted drug delivery to the brain. FUS-BBBO is also capable of modulating the neuroimmune system, further qualifying its therapeutic potential for neurodegenerative diseases like Alzheimer’s disease (AD). Natural aging and AD impose significant strain on the brain and particularly the BBB, modifying its structure and subsequently, its functionality. The emerging focus on treating neurodegenerative diseases with FUS-BBBO necessitates an investigation into the extent that age and AD affect the BBB’s response to FUS. FUS-BBBO was performed with a 1.5-MHz, geometrically focused transducer operated at 450 kPa and paired with a bolus microbubble injection of 8 × 10^8^ μB/mL. Here we quantify the BBBO, BBB closing (BBBC) timeline, and BBB permeability (BBBP) following FUS-BBBO in male mice with and without AD pathology, aged 10 weeks, one year, or two years. The data presented herein indicates that natural aging and AD pathology may increase initial BBBO volume by up to 34.4% and 40.7% respectively, extend BBBC timeline by up to 1.3 and 1.5 days respectively, and increase BBBP as measured by average K^trans^ values up to 80% and 86.1% respectively in male mice. This characterization of the BBB response to FUS-BBBO with age and AD further clarifies the nature and extent of the functional impact of these factors and may offer new considerations for planning FUS-BBBO interventions in aged and AD populations.

## Introduction

The blood–brain barrier (BBB) is a tightly-regulated organization of endothelial cells (ECs), pericytes (PCs) and astrocyte end feet that surrounds cerebral vasculature and mediates material exchange between the brain and circulatory system^1,2^. ECs are tightly bound together by tight junction (TJ) proteins that regulate paracellular transport across the barrier, and are surrounded by a layer of PCs that secrete supportive chemical factors such as TGFβ and VEGF^2,3^. Astrocyte end feet further support the barrier by physically encircling the cerebral vessels and enabling bulk fluid flow via aquaporin 4 expression^2^. ECs, mural cells, immune cells, glia and neural cells all interact at the neurovascular unit (NVU) to support and maintain the BBB^1^. In healthy subjects this coordinated multi-cellular barrier acts as a mediator between the brain and systemic vasculature, constructively preventing the entry of deleterious species into the brain, but inhibiting the passage of all large molecule drugs and 98% of small molecule drugs^4,5^. This highly-restrictive permeability protects the central nervous system (CNS) from circulating pathogens, albeit introducing a significant obstacle in drug delivery to the CNS^4^.

The progression of age imposes significant strain on the BBB, detracting from the health, structure and function of the cells that make it up^6^. Age-associated cortical thinning, ventricle enlargement, and increased BBB permeability have been reported in mouse and human tissue^7–10^. Investigations into the mechanism of increased cerebral permeability overwhelmingly implicate the degradation of TJ proteins, caused by cellular senescence, the presence of plaques, and signaling by various disease- and age-related factors^2,11,12^. In addition to the structural, synaptic and neural changes that occur, age may also evoke a decrease in cerebral blood vessel density, endothelial dysfunction, arterial stiffening, and stunted vascular repair, all of which detract from the health of the NVU and may affect the brain’s response to FUS-BBBO^2,13^. In addition to natural aging, brain pathologies, such as Alzheimer’s disease (AD), also diminish NVU and CNS health^8^.

Alzheimer’s disease is a neurological, age-associated disease that affects over 50 million people worldwide^14^. AD is responsible for 60–70% of all cases of dementia, and has an estimated 10 million new diagnoses every year^15^. Clinical symptoms of AD include diminished episodic memory, difficulty multi-tasking, and a mild cognitive impairment (MCI) diagnosis^16^. Pathologically, AD is characterized by the accumulation of extracellular Amyloid-β (Aβ) plaques and intracellular neurofibrillary tau tangles. This inappropriate protein build-up results in neurodegeneration, atrophy and synaptic loss^17^. The hippocampus and neocortex, two brain regions heavily implicated in learning and memory, are the most significantly affected in early stage AD^17^. BBB permeability as measured by K^trans^ has been shown to increase in humans with MCI or AD, thereby demonstrating accelerated BBB degradation in the presence of AD pathology^9,18^.

The triple transgenic (3xTg) AD mouse model recapitulates both pathological and cognitive hallmarks of human AD^19–21^. This model harbors PS1_M146V_, APP_Swe_, and tau_P301L_ mutations, which give rise to the progressive accumulation of Aβ plaques, tau tangles, and synaptic dysfunction^19^. APP and Aβ filaments appear in the hippocampus by 3 months of age, however Aβ40 and Aβ42 accumulation are detectable only after 12–15 months of age in the hippocampus and cortex of male 3xTg mice^22,23^. Phosphorylated and total tau are detectable in male mice at 4–6 months of age^22,23^. With age and the accumulation of pathology comes the worsening of cognitive and behavioral symptoms. By the age of 18 months, male 3xTg mice exhibit inhibited long-term potentiation, although impairments in spatial memory and cognition appear earlier, by 6 months of age^23,24^. Age-dependent changes in transport mechanisms at the BBB, progressive microvascular degeneration, increased cortical vascular density, and impaired neurovascular coupling due to cerebrovascular dysfunction have also been demonstrated in 3xTg mice^25–29^. The expression of TJ proteins has also been shown to decrease with age in 3xTg mice^30^. Given the direct importance of BBB integrity and vasculature in the brain for determining the effects of Focused Ultrasound-induced BBBO (FUS-BBBO), the characterization of these structural changes motivates an investigation into their influence on the effects of FUS-BBBO in mice.

Focused Ultrasound is a noninvasive technique that involves focusing acoustic pressure within a targeted region in the brain. The focused pressure waves interact with systemically-introduced, lipid-shelled, gas-filled microbubbles (μB) passing through the focal volume, causing them to cavitate and mechanically disrupt the surrounding vascular lining, loosening intercellular TJ proteins, increasing endothelial cell permeability, and transiently opening the BBB^31,32^. FUS-BBBO presents an attractive technique to circumvent the highly-selective permeability of the BBB by opening the barrier to larger agents in a reversible, noninvasive and spatially-precise manner.

The safety of FUS-BBBO has been validated in many animal models, including mice, rabbits, sheep and nonhuman primates (NHPs)^33–35^. Studies conducted primarily in young mice have reported barrier closing times ranging from 10 minutes to five days depending on the specific parameters used^36–38^. The application of FUS has also proven efficacious for eliminating AD pathology and improving behavioral deficits in murine models of neurodegenerative disease^39–41^. Increased neurogenesis, enhanced long-term potentiation, AD-associated protein reduction (Aβ and tau), as well as improved cognition and spatial memory have been demonstrated with FUS-BBBO in murine models of AD^40–44^. FUS-BBBO has also been shown to trigger a dynamic neural immune response, particularly in microglia and astrocytes^39,45–47^. Theories about the mechanism behind the observed reduction in pathology primarily implicate increased phagocytosis by resident immune cells or enhanced lymphatic drainage following FUS-BBBO^39,48^. The demonstrated safety of this technique and its effectiveness in preclinical studies for AD protein reduction and symptom amelioration have motivated and enabled translation into the clinic, where human trials are actively underway^49–51^.

Growing interest in the use of FUS-BBBO for the treatment of neurodegenerative disease in aged populations motivates an investigation into the effects of natural aging and AD pathology on the brain’s response to FUS-BBBO. More specifically, the present study aims to characterize the initial volume of BBBO, the duration of time required for BBB reinstatement, and the permeability of brain tissue within the volume of the BBBO with the progression of age and AD to inform the functional effects of these factors in the context of FUS-BBBO.

## Results

### Cavitation dose does not vary significantly between cohorts

**Figure 1:**
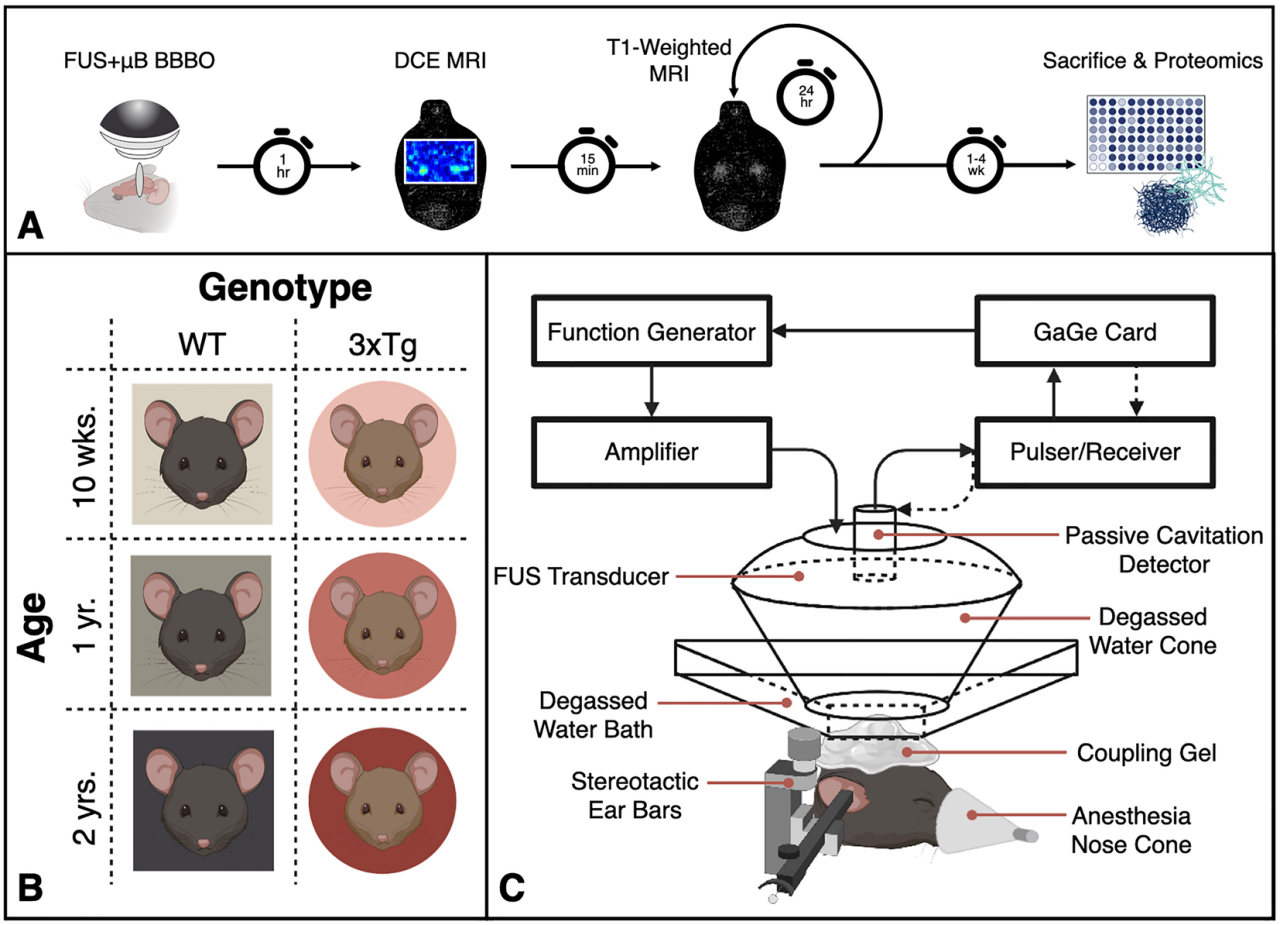
Experimental design. (**A**) Overview of experimental timeline and workflow. (Elements created with BioRender.com) (**B**) Experimental cohorts used throughout the study. WT and 3xTg mice aged 10 weeks, one year or two years were used for the present study, with 5–9 animals per group. (Created with BioRender.com) (**C**) Schematic of Focused Ultrasound setup. (Created with BioRender.com).

**Figure 2:**
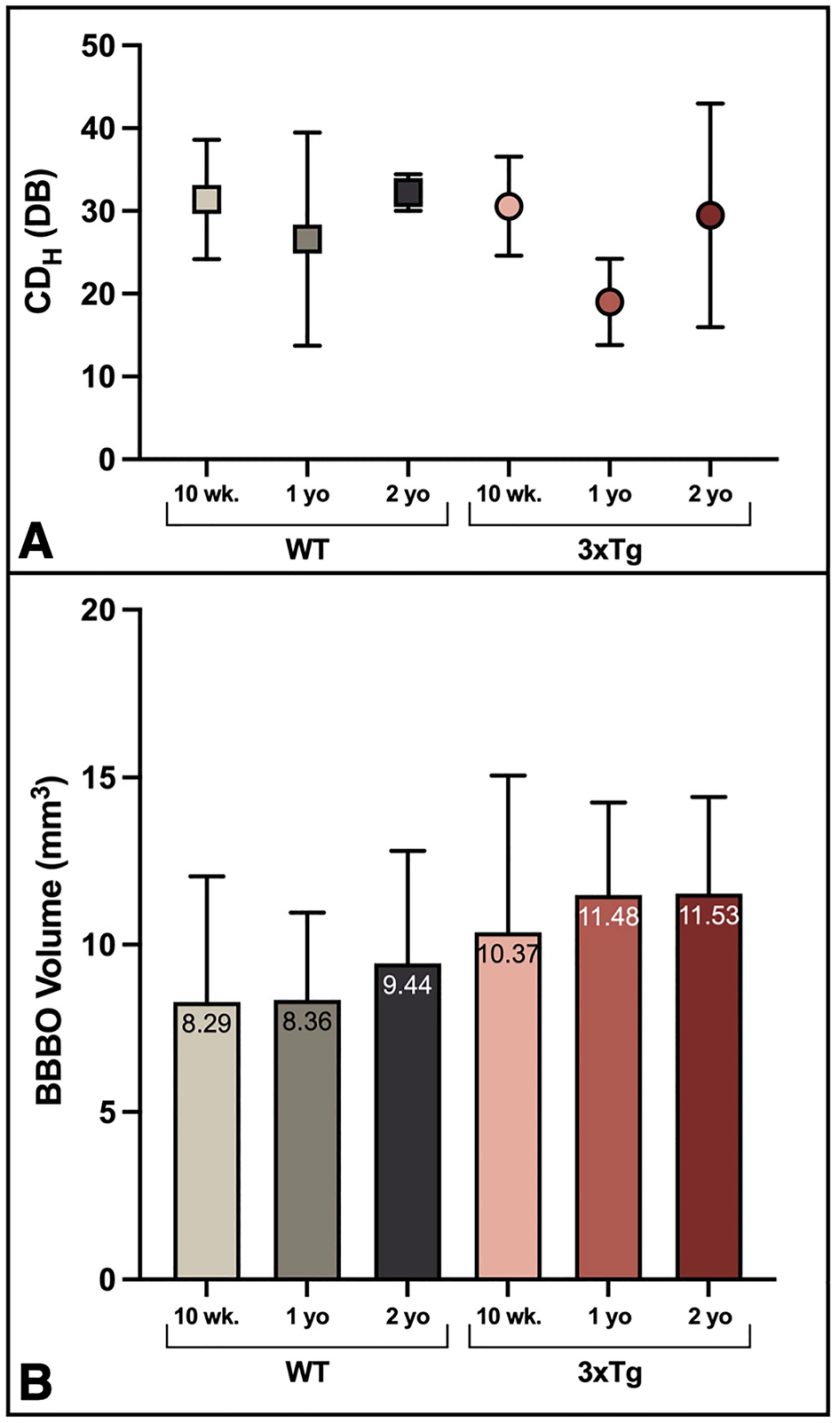
Age and AD produce trending increase in BBBO volume with consistent stable harmonic cavitation dose. (**A**) The mean stable harmonic cavitation dose (CD_H_) delivered to each group is shown. No significant differences were detected between groups (*P* > 0.05, one-way ANOVA with multiple comparisons). (**B**) The average initial BBBO volume for each cohort is shown. Differences between groups are not significant (*P* > 0.05, one-way ANOVA with multiple comparisons).

Figure 1A shows the experimental workflow for FUS-BBBO in male mice aged 10 weeks, 1 year, and 2 years with either WT or 3xTg genotypes (Fig. 1B). The emissions from microbubble activity can be measured and quantified to serve as a proxy for the dose of FUS administered. The stable, harmonic cavitation dose (CD_H_) measured for each mouse and each cohort on average did not differ significantly between groups (Fig. 2A) (*P* > 0.05, one-way ANOVA with multiple comparisons). This result is expected based on the use of equivalent microbubble doses and sonication parameters between groups. Variations between mice are within the error margin of the passive cavitation detector (PCD) and are not the result of significant experimental differences. Still, confirmation of this equivalent dose is necessary for all subsequent analysis, which assumes consistent treatment across all mice.

### Age and AD elicit trending increase in BBB opening volume

**Figure 3:**
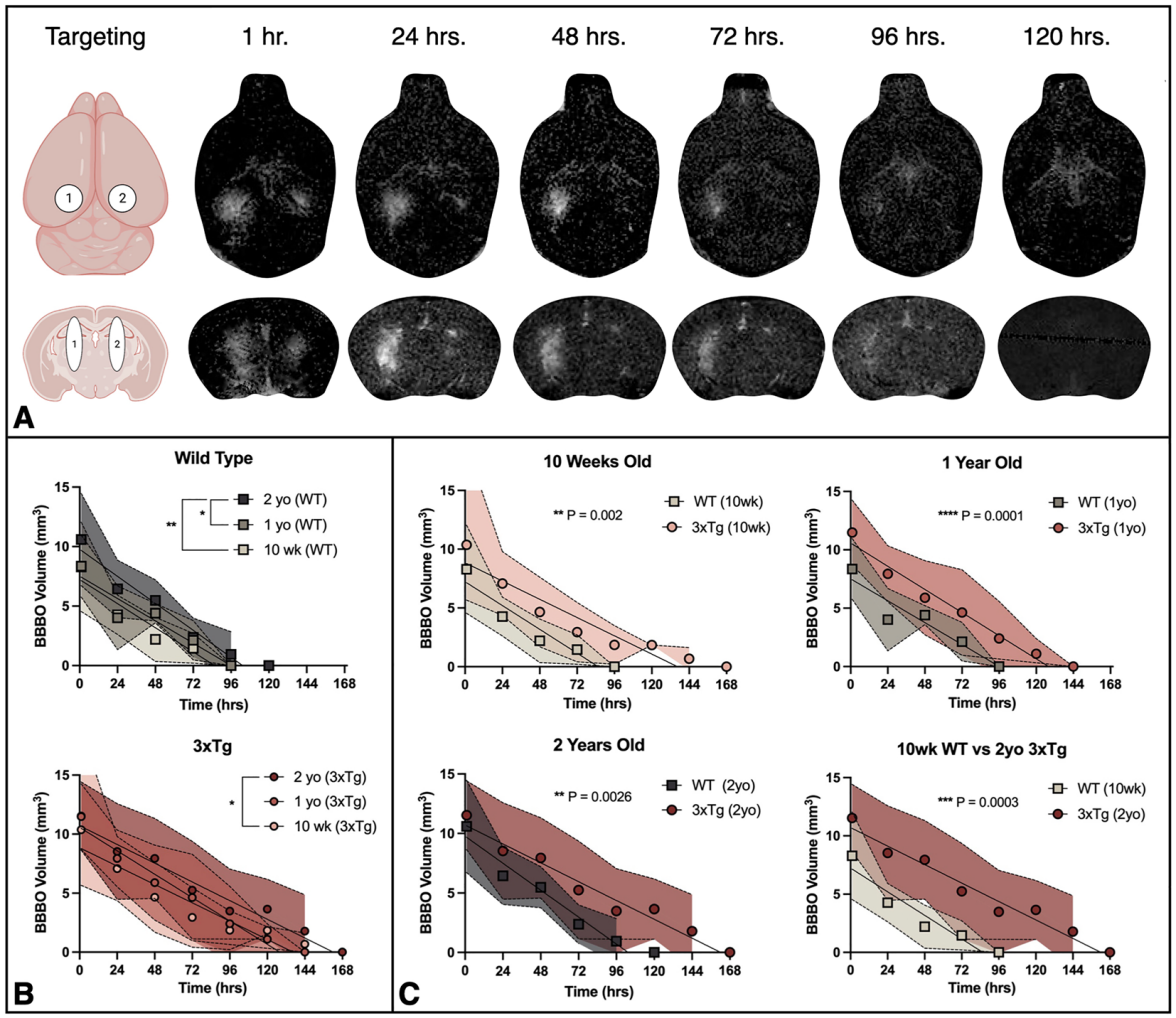
Linear regression of blood–brain barrier opening volume over time reveals increased fitted initial opening volume with age and AD. (**A**) BBBO targeting and representative MR axial and coronal images acquired at 24-h intervals after FUS-BBBO show the daily BBBO volume and progressive closing. (Elements created with BioRender.com) (**B**) Average BBBO volume and standard deviation is plotted as a function of time for WT and 3xTg cohorts (**B**) as well as for 10-week-old, one-year-old, and two-year-old cohorts (**C**). Linear regression analysis and statistical testing by ANCOVA was used to detect significant differences between fitted y-intercepts.

**Figure 4:**
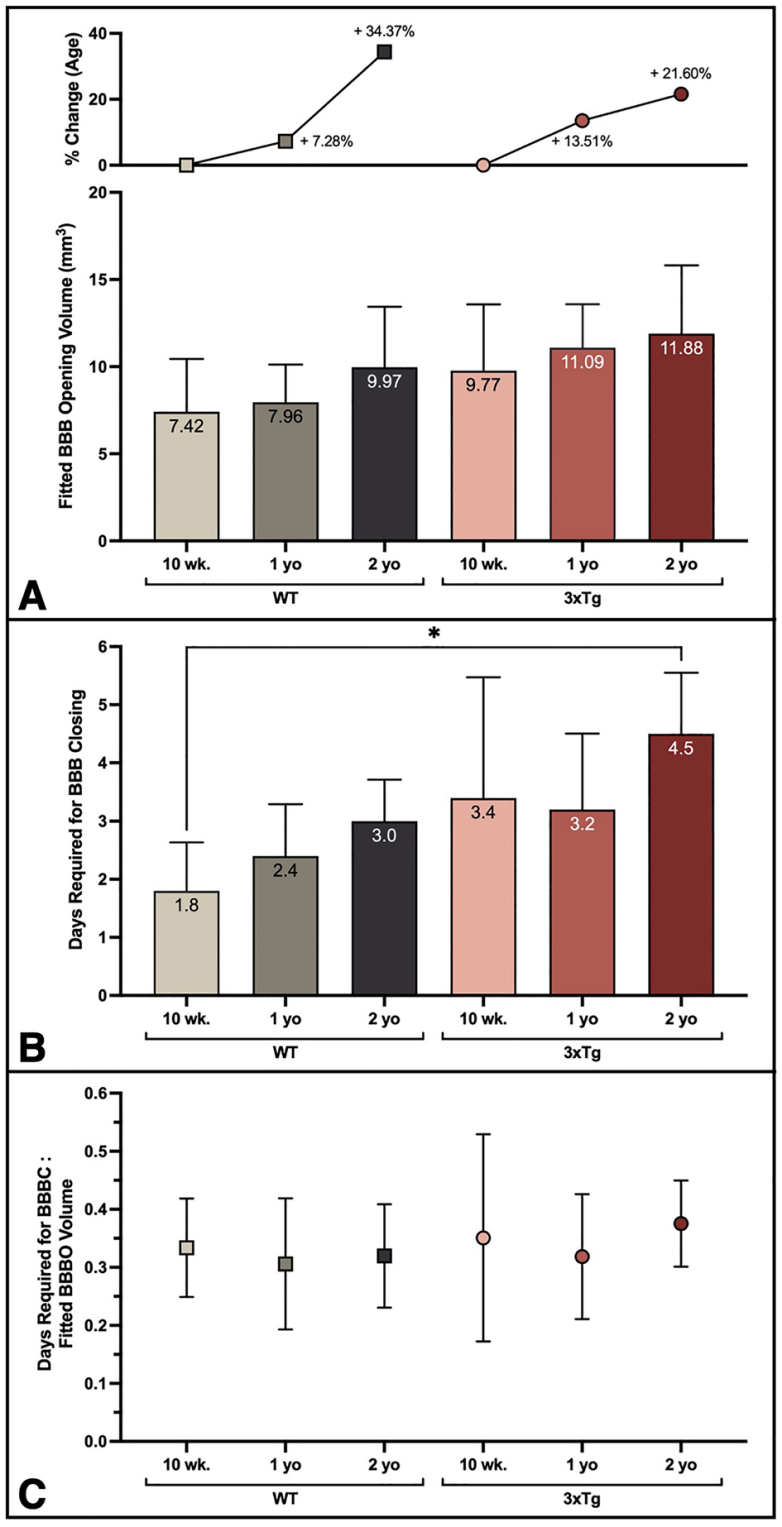
Age and AD elicit trending increase in fitted BBBO and prolonged BBBC timeline. (**A**) The average fitted y-intercept from individual linear regression plots of subjects from each cohort is plotted. The percent change for one- and two-year old cohorts of each genotype compared to the respective 10-week-old cohort is shown in the top plot. (**B**) The average time required for BBB closing following FUS-BBBO is shown for each cohort. (**C**) The ratio of the time required for BBBC to the fitted initial BBBO volume is shown for each cohort. Statistically significant differences are determined by one-way ANOVA with multiple comparisons.

Gadolinium (Gd) diffusion across the BBB following FUS-BBBO can be visualized with MRI, and shows the volume of BBBO in vivo (Fig. 3A). Quantification of the hyperintense volume of Gd diffusion serves as a measure of the initial BBBO in each animal. On average, quantification of this initial BBBO volume revealed a non-significant but trending increase with age and AD pathology (Fig. 2B). Although the initial, measured volume of BBBO was not significantly different between cohorts (*P* > 0.05, one-way ANOVA with multiple comparisons), there is a trending increase in the initial opening volume with age and AD. In WT mice, the average opening volume increased from 8.29 mm^3^ in 10-week-old WTs to 9.44 mm^3^ in 2-year-old WTs. In 3xTg mice the trend was the same, increasing from 10.37 to 11.53 mm^3^ in the 10-week-old compared to 2-year-old 3xTg cohorts. Additionally, pairwise, age-matched comparisons consistently demonstrate greater average BBBO volumes in 3xTg cohorts compared to the age-matched WTs. The volume of BBBO was plotted as a function of time after FUS-BBBO (Fig. 3B, C) and was regressed to fit a linear line for each group. The y-intercepts of these lines were then interpreted as the fitted initial opening volume for the cohort. When comparing the y-intercepts of each group, significant differences were detected. Genotype-matched cohorts demonstrated increased fitted opening volumes with age (Fig. 3B). In WT mice, the two-year-old cohort had a significantly greater fitted opening volume of 9.770 mm^3^ compared to the 10-week-old volume of 7.234 mm^3^ (*P* = 0.0038, ANCOVA) and one-year-old volume of 7.513 mm^3^ (*P* = 0.0295, ANCOVA) (Fig. 3B). In 3xTg mice, the two-year-old cohort had a significantly greater fitted initial opening volume of 10.71 mm^3^ compared to the 10-week-old volume of 8.848 mm^3^ (*P* = 0.0278, ANCOVA) (Fig. 3B). Comparison of age-matched cohorts revealed a statistically significant increase in BBBO volume in 3xTg compared to WT at 10 weeks (*P* = 0.002, ANCOVA), one year (*P* = 0.0001, ANCOVA), and two years old (*P* = 0.0026, ANCOVA) (Fig. 3C). Finally, comparison of the 10-week-old WT cohort to the two-year-old 3xTg cohort demonstrated a significant, 3.476 mm^3^ increase in BBBO with both age and AD (*P* < 0.0003, ANCOVA) (Fig. 3C). Linear regression equations for each cohort and associated R^2^ values are shown in Table 1. In addition to interpreting the y-intercepts as the fitted opening volume for each cohort, the volume of BBBO was plotted for each mouse as a function of time and the y-intercept was regressed for each individual. These individual fitted y-intercepts were averaged for each group and are shown in Fig. 4A. Compared to 10-week-old WT animals, 1- and 2-year-old fitted BBBO volumes increased by 7.28% and 34.37% respectively (Fig. 4A). In 3xTg animals, 1- and 2-year-old cohorts had 13.51% and 21.60% greater fitted BBBO volumes compared to the 10-week-old 3xTg cohort respectively. There was no statistically significant difference between fitted BBBO volumes for all six groups by one-way ANOVA with multiple comparisons (*P* > 0.05). However, pairwise, unpaired t tests between groups revealed significant increases in BBBO volume in the one-year-old 3xTg cohort compared to both the 10-week-old and one-year-old WT cohorts (*P* = 0.0304 & *P* = 0.0369 respectively, Unpaired t test). Taken together, the increasing trend in these results indicates that both the progression of age and AD may slightly increase the extent of initial BBBO in response to equivalent sonication parameters.

**Table 1:** Linear regression results for BBB closing plots.

Age	Genotype	Linear regression equation	R^2^
10 wk.	WT	Y = − 0.08446*X + 7.234	0.6071
	3xTg	Y = − 0.06478*X + 8.848	0.5497
1 yr.	WT	Y = − 0.07812*X + 7.513	0.6163
	3xTg	Y = − 0.08258*X + 10.57	0.5953
2 yr.	WT	Y = − 0.09442*X + 9.770	0.6785
	3xTg	Y = − 0.06604*X + 10.71	0.4897

### Age and AD may extend BBB closing timeline

The amount of time required for BBBC was extended by both advanced age and AD pathology. The slopes of the linear regression lines fitted to BBBC profiles (Fig. 3B, C) can be interpreted as the rate of BBBO reinstatement. Although the slopes of the BBBC regression lines did not vary significantly, the amount of time required for repair was extended significantly with age and AD progression (Fig. 4B). Comparison of the number of days required for BBB closing reveals that two-year-old 3xTg mice required significantly more time for closing than 10-week-old WT mice (*P* = 0.0140, one-way ANOVA with multiple comparisons) (Fig. 4B). Although differences between other groups are not statistically significant, there is a clear increasing trend in the time required for closing with age in both WT and 3xTg cohorts, as well as with AD when comparing age-matched cohorts. The ratio of the days required for BBBC to the fitted BBBO volume does not vary significantly between groups by one-way ANOVA with multiple comparisons (Fig. 4C).

Taken together, these data suggest that the time required for BBB reinstatement may be extended by age and AD progression, however that the initial BBBO volume might be the driving factor for this extension given the proportionality of the ratio of BBBC time to BBBO volume across all six groups in Fig. 4C.

### Baseline permeability to gadolinium does not vary significantly between cohorts

The mean and maximum K^trans^ permeability values calculated within a cortical, unsonicated control volume for each mouse revealed no significant difference in the permeability of these control tissues to Gd across age and genotype cohorts (Fig. S1A , B, one-way ANOVA with multiple comparisons). A heat map of the average K^trans^ map for each cohort is shown in Fig. S1C as a representation of the background K^trans^ map in a control region of the brain.

### Age and AD increase the measured K^trans^ permeability coefficient in BBBO region

**Figure 5:**
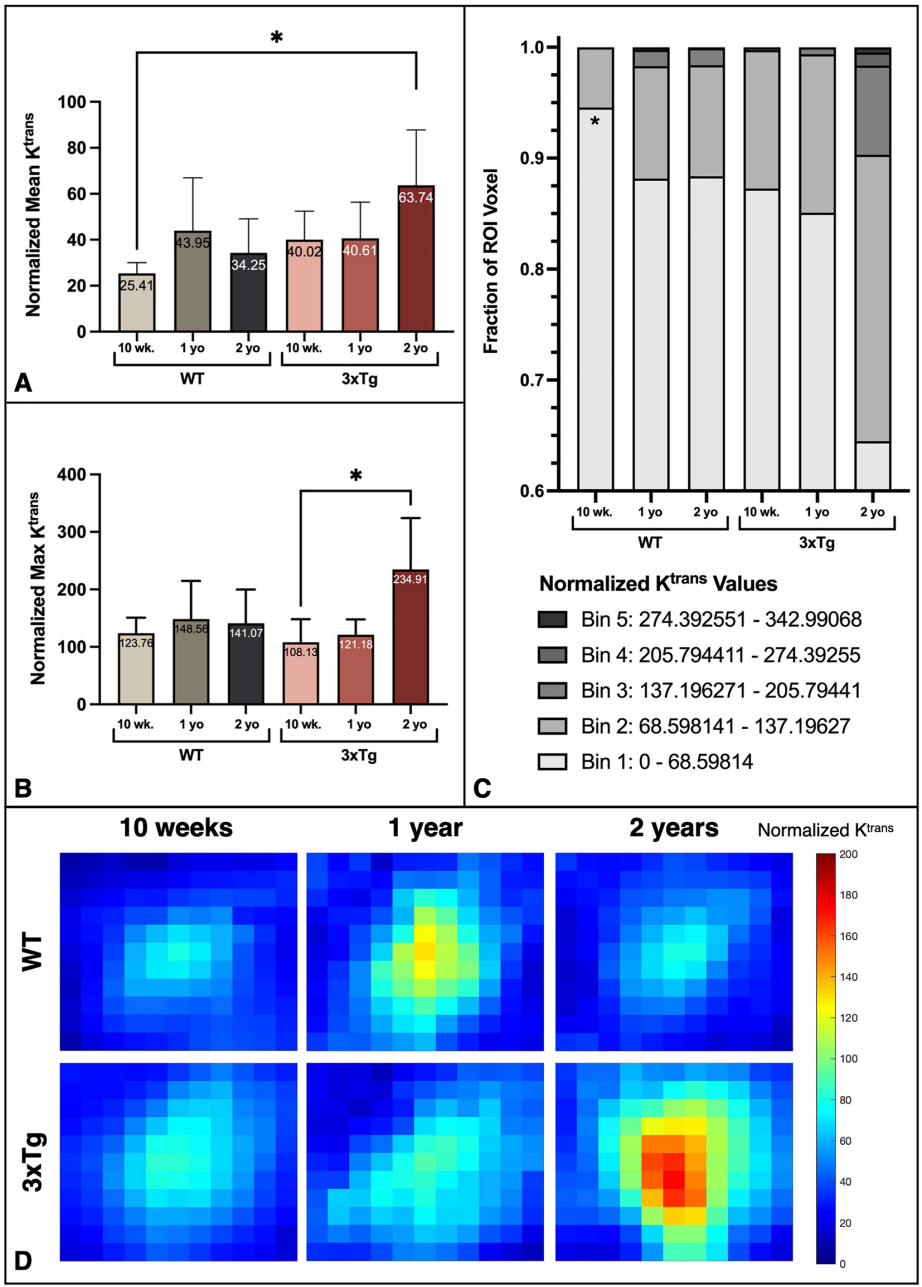
Age and AD increase the measured mean and maximum K^trans^ permeability coefficients in BBBO region. (**A**) The mean K^trans^ value normalized by mean background value for each cohort is shown. (**B**) The normalized maximum K^trans^ values are shown for each age and genotype group. (**C**) The average fraction of the ROI volume within the specified bin range is shown for each group. Significant differences from the two-year-old 3xTg cohort are indicated by asterisks. All statistically significant differences were determined by one-way ANOVA with multiple comparisons for (**A**–**C**). (**D**) The average 2D K^trans^ map from the middle slice of an ROI volume encompassing the BBBO focal volume is shown for each cohort. K^trans^ maps have been normalized by baseline K^trans^ values.

After normalizing each K^trans^ map by the mean background K^trans^ value for each cohort, the average K^trans^ value within the BBBO volume was significantly higher in the two-year-old 3xTg compared to the 10-week-old WT cohort (*P* = 0.0379, one-way ANOVA with multiple comparisons) (Fig. 5A). The maximum normalized K^trans^ values also increased with age and AD, with the two-year-old 3xTg group reaching significantly greater values than the 10-week-old 3xTg cohort (*P* = 0.0404, one-way ANOVA with multiple comparisons) (Fig. 5B). In addition to these increasing global values, the fraction of the ROI volume made up of K^trans^ values belonging to the lowest Bin 1 of values was significantly higher in the 10-week-old WT cohort than in the two-year-old 3xTg cohort (*P* = 0.0450, one-way ANOVA with multiple comparisons) (Fig. 5C), indicating that lower K^trans^ values dominated the ROI volumes of these groups. Out of all six cohorts, the two-year-old 3xTg cohort had the smallest fraction of the ROI volume made up of low, Bin 1 K^trans^ values. The two-year-old 3xTg cohort also had the greatest fraction of the ROI volume made up of the higher K^trans^ values in Bins 2, 3, 4 and 5 compared to all other cohorts. Taken together, these data indicate that age and AD increased the K^trans^ values within the BBBO volume and skewed the distribution toward higher K^trans^ values. The average K^trans^ heat map encompassing the BBBO region for each cohort is shown in Fig. 5D.

Pooling cohorts by age and genotype further clarifies the impact of these factors on K^trans^ permeability. When mice of both genotypes are pooled by age, there was an insignificant trending increase in the normalized mean K^trans^ value with increased age (Fig. S2A). The mean K^trans^ value also increases without significance in the pooled 3xTg group compared with pooled WT mice (Fig. S2B). The maximum K^trans^ value within the BBBO volume also demonstrated a trending increase with age (Fig. S2C) and AD (Fig. S2D).

The comparison across all six cohorts and between age- and genotype-matched groups consistently demonstrated increased average and maximum K^trans^ values within the 3D ROI volume encompassing the BBBO volume with age and AD.

### Concentration of AD-associated proteins increases with age in 3xTg cohorts

The total protein-normalized concentration of four AD-associated proteins increased with age in the 3xTg mice used in this study. The mean, normalized concentration of Aβ40 increased with age from 9.951 × 10^−7^, to 1.159 × 10^−6^, to 1.494 × 10^−6^ in 10-week-, one-year-, and two-year-old cohorts respectively (Fig. S3A). The normalized Aβ40 concentration was significantly higher in the two-year-old cohort compared with 10-week-old mice (*P* = 0.0349, one-way ANOVA with multiple comparisons) (Fig. S3^3^). This trend continues with the total protein-normalized concentration of Aβ42, which was significantly higher in two-year-old 3xTg mice compared to 10-week- (*P* = 0.0005) and one-year-old (*P* = 0.0080) mice by one-way ANOVA with multiple comparisons (Fig. S3B). Although not significant, there was a trending increase in the concentrations of pT181 (Fig. S3C) and Total Human Tau (Fig. S3D) with age across the 3xTg cohorts. This increase in pathology is consistent with the expectation for the 3xTg-AD model but validation enables claims made about the effects of disease progression on the BBB properties discussed.

## Discussion

Many studies characterizing the effects of FUS-BBBO have been conducted in young WT mice, however the emerging interest in treating neurodegenerative disease in aged populations with FUS-BBBO has motivated the present study, evaluating the effects of natural aging and AD pathology on the brain’s response to FUS-BBBO.

Here we show that despite the unchanging FUS treatment parameters across groups, there is a clear trending increase in the volume of Gd diffusion to the hippocampus, which we interpret as an increase in the volume of BBBO, following FUS-BBBO with age and AD pathology. Although differences between the measured BBBO volume between groups one hour following FUS-BBBO were not statistically significant, the increasing trend is clear. Additionally, the static MRIs acquired one hour after Gd injection may not offer the most accurate measure of the BBBO volume due to the delay in acquisition, one hour after injection. The hour required for the DCE scan significantly delays the acquisition of the static T1-weighted MRI. This delay enables the gadolinium to diffuse beyond the initial opening volume, narrowing the measurable differences between cohorts as a result of the constant volume of Gd injected into each mouse. On subsequent days, the static T1-weighted MRI was acquired 15–20 min after Gd injection, allowing the Gd sufficient time to enter the BBBO volume, without diffusing beyond into surrounding tissue. This metric makes the fitted y-intercept of the linear regression closing timeline a better estimate of the initial opening volume than the delayed, static T1-weighted MRI acquired on the first day. Thus, the statistically significant increases in fitted BBBO volumes by cohort (Fig. 3B-C) and increasing trend in individual linear regression (Fig. 4A) better represent the age- and AD-associated increases in initial BBBO volumes. Although the focal volume of the transducer remains unchanged for all subjects, the increase in BBBO volume can be interpreted as the result of increased susceptibility to opening in aged, AD mice. Thus, the consistent increase in fitted BBBO volumes with age and AD indicates that aged and pathological brain tissue had the lowest threshold for BBBO. This finding is consistent with our expectation based on previous studies showing compromised BBB integrity with age and AD due to increased breakage or decreased expression of TJ proteins by degenerating ECs^3,10,30,52–55^. The previously characterized negative correlation between TJ protein expression and AD progression in both humans and 3xTg mice offers a potential mechanistic explanation for the observed negative relationship between BBBO threshold and AD progression in the present study^30,55^. Both age and AD were found to increase the initial BBBO volume, as demonstrated by the increase detected in the fitted initial BBBO volume in aged and 3xTg cohorts compared to genotype- and age-matched cohorts in Fig. 3B, C.

Although the fitted initial BBBO volume is significantly increased with age and AD progression, the lack of significant differences between the slopes of each cohort’s BBBC linear fit indicates that the rate of BBBC after FUS-BBBO is not significantly affected by age or AD progression. Instead, the extended closing timeline with age and AD, as shown in Fig. 4B, may be a consequence of the larger initial opening volumes observed in these groups. This hypothesis is further reinforced by the lack of significant differences in the ratio of time required for BBBC to the fitted BBBO volume between groups (Fig. 4C). In other words, the time required for BBBC is proportional to the initial BBBO volume in each group. While the mechanisms of BBB reinstatement are still under active investigation, the data presented herein suggests that the mechanism of BBBC following FUS-BBBO may not be significantly compromised by age or AD pathology, but rather that BBBO volume may be a better predictor of BBBC timelines. Additionally, it’s been shown that FUS is capable of activating neural immune cells, especially astrocytes and microglia, which likely play a crucial role in BBB reinstatement^39,56,57^. Thus, aged and young neuroimmune cells alike may be capable of responding to such stimulation by FUS-BBBO to reinstate the barrier at a rate that is proportional to the initial opening volume.

Analysis of a control volume in the cortex, outside of the BBBO volume, reveals that neither the mean nor maximum K^trans^ values varied significantly between cohorts. Previous studies comparing BBB permeability in aged and AD brains to healthy controls report mixed results depending on the measure of permeability^9,53,58,59^. This discrepancy motivated the use of the measured background K^trans^ values as a normalization factor for the K^trans^ values measured in the focal region. This normalization justifies the conclusion that any differences in K^trans^ detected within the BBBO focal volume are the direct result of the tissue’s response to FUS-BBBO, and not a consequence of the baseline physiology. Trending increases in mean and maximum K^trans^ values were observed within the BBBO volume with both age and AD. Additionally, the distribution of K^trans^ values varied across the BBBO volume between aged and AD cohorts. The aged and AD cohorts are dominated by higher K^trans^ values, while the younger and WT cohorts are skewed more toward lower K^trans^ permeability values. A larger fraction of the ROI volume made up of higher K^trans^ values is indicative of a larger affected area from FUS-BBBO, further demonstrating the more extensive response to FUS-BBBO in aged and AD mice. Grouping cohorts into age- and genotype-matched groups in Fig. S2 further reveals the effects of age and AD, consistently confirming that both age and the presence of AD pathology increase tissue permeability within the BBBO volume, as measured by K^trans^ mean and maximum values. Previous studies have reported reduced vessel permeability to fluorescent dextran from in-tact vessels in pathological brains compared to WT using two-photon imaging^60^. However, these findings were measured only from in-tact vessels, omitting those that were leaky prior to FUS application, which may comprise a significant percentage of vessels in the opening volume within AD brains. Additionally, the kinetics of dextran leakage are expected to differ from that of gadolinium as used in the present study. The overall permeability to gadolinium was found to increase with age and AD in the present study as measured by an ROI encompassing the focal volume, consistent with previous reports of increased permeability in the hippocampi of individuals with age and AD pathology^9,53,61^.

## Conclusion

Many foundational studies characterizing FUS-BBBO have been conducted in young, healthy mice, necessitating a study on its effects in aged and AD cohorts that more closely represent the target patient population. In this study, we demonstrated that natural aging and the progression of AD pathology may increase the brain’s susceptibility to FUS-BBBO. Age and AD were observed to amplify the effects of FUS-BBBO by slightly increasing BBBO volume and BBBP, and extending the timeline required for BBBC. The trends reported here may offer important considerations for clinical pre-planning and therapeutic design of FUS-BBBO interventions in aged and AD populations. Currently, patient-specific features such as skull thickness and geometry are incorporated into simulations that inform treatment planning for FUS-BBBO in humans. The results of this study indicate that perhaps additional personalized metrics, such as tissue health and baseline BBB permeability, should also be considered while tuning FUS parameters for individual patients treatment planning. Taking into consideration individualized features that influence the effects of FUS-BBBO will make translation of FUS-BBBO safer and more effective in human subjects.

## Materials and methods

### Study design

This study was conducted exclusively in mice in compliance with protocols approved by Columbia University’s Institutional Animal Care and Use Committee. All methods reported herein are compliant with ARRIVE guidelines. Each subject underwent a single session of FUS-BBBO, bilaterally targeting the hippocampus. Each subject was administered an IP injection of Gd contrast agent, then underwent dynamic contrast-enhanced (DCE) magnetic resonance imaging (MRI) to measure BBBP, followed by a static T1-weighted MRI to visualize the BBBO one hour after treatment with FUS + μB. Every 24 h following FUS-BBBO the subjects received a new Gd injection, and a static T1-weighted MRI was acquired to track the BBBC timeline. Once the BBB was closed, as determined by the absence of signal enhancement on T1-weighted MRI following Gd injection, the animals were sacrificed, and the brain tissue was collected for protein quantification (Fig. 1A).

### Animals

All animals were housed and handled in accordance with Columbia University’s Institutional Animal Care and Use Committee regulations. 35 male mice were used for the present study, divided between six groups varying in age and genotype. These groups included wild type (WT) and triple transgenic Alzheimer’s disease model 3xTg mice (Strain #004,807, MMRRC stock #034,830-JAX, The Jackson Laboratory, Bar Harbor, ME, USA), aged ten weeks, one year, or two years (n = 5–9/group) (Fig. 1B)^19^. These ages were selected to represent young, middle-aged and elderly subjects, with early, moderate and late-stage AD progression in 3xTg cohorts. Phenotypic color differences between WT and 3xTg mice precluded full experimental anonymization of each subject’s cohort, however subject treatment order was randomized throughout the study to prevent biases or batch effects.

### Focused ultrasound + microbubbles

FUS-BBBO was performed as previously described^32^. Briefly, a single-element, concave FUS transducer (center frequency: 1.5 MHz, focal length: 60 mm, diameter: 60 mm, Imasonic, France) was operated at a Peak Negative Pressure of 450 kPa with a pulse repetition frequency of 4 Hz. An additional single-element transducer (V320, frequency: 7.5 MHz, focal length: 52 mm, diameter: 13 mm; Olympus NDT, Waltham, MA) was confocally aligned with the FUS transducer focus and used for passive cavitation detection. These transducers were fixed to a 3D positioning system for alignment and targeting. Subjects were anesthetized with 3–4% isoflurane and sustained at 1.5% isoflurane via a nose cone while stereotactically stabilized for FUS-BBBO. Fur was removed from the top of the head using electric clippers and depilatory cream, and coupling gel was applied between the head and water bath in which the FUS transducer was submerged. This FUS setup is shown in Fig. 1C. The transducer was moved via the 3D positioning system, to each target location on the hippocampus, 2 mm rostral and 1.8 mm lateral to the lambdoid structure (Fig. 3A). Prior to injection of microbubbles, 10 control pulses were recorded at each bilateral target for later cavitation dose calculation. Once the control pulses were recorded, 5 μL activated in-house made polydisperse microbubbles were diluted in 45 μL sterile saline and injected intravenously in a bolus dose of 8 × 10^8^ μB/mL, immediately prior to activation of the FUS transducer. Subjects were sonicated for 60 s per bilateral target, for a total sonication time of two minutes. Bubble cavitation was recorded throughout sonication. Identical sonication parameters were used across all mice.

### Magnetic resonance imaging

#### DCE T1-weighted MRI

DCE MRI is an MRI sequence used to estimate the permeability of tissue barriers based on fitted estimates of the material transfer rate K^trans^^62^. This technique involves the acquisition of a time course of DCE MR images both before and after intraperitoneal injection of Gd contrast agent. These time-course images are then fitted to a general, two-compartment kinetic model consisting of blood plasma and extracellular extravascular space^62^. The rate of exchange of Gd between these two compartments is reflected in the fitted transfer constant K^trans^, and the K^trans^ map generated for each mouse^62,63^. These maps then provide a quantitative representation of the permeability across a two-dimensional slice of brain tissue by measuring the rate of Gd flow into the brain. A z-stack of these MRI slices can then be compiled and interpolated to perform permeability analysis over a 3D volume. Each subject received an intraperitoneal catheter and was set inside of the MRI (Bruker Ascend™ 400MHZ WB 9.4 T). A DCE sequence (TR: 40 ms, TE: 1.4 ms, Flip angle: 50°, Averages: 6, Repetitions: 55, FOV: 25.6 mm × 25.6 mm, Matrix size: 160 × 160, Slice Thickness: 0.6 mm, Resolution: 0.16 mm × 0.16 mm, Scan time: 35 min) was then initiated and four reference frames were acquired. 0.3 mL of Gd contrast agent was injected into the catheter followed by a saline flush, and a time series, z-stack of axial MR images was acquired to track Gd diffusion into the BBBO volume.

#### Static T1-weighted MRI

Approximately one hour after FUS-BBBO, following the DCE sequence a static, contrast-enhanced, T1-weighted 2D FLASH sequence (TR: 230 ms, TE: 3.3 ms, Flip angle: 70 deg, Averages: 6, FOV: 25.6 mm × 25.6 mm, Matrix size: 256 × 256, Slice thickness: 0.4 mm, Resolution: 0.1 mm × 0.1 mm, Scan time: 5 min) was acquired in the axial and coronal directions for targeting confirmation and later BBBO volume quantification.

24 h later the subjects received an additional IP injection of 0.2 mL Gd contrast agent and were rescanned 15 min after injection with a T1-weighted 2D FLASH sequence for BBBO volume quantification. This procedure was repeated daily until Gd contrast agent was no longer visible by MRI, indicating BBB closure (Fig. 3A).

### Data processing

#### Cavitation dose

The stable, harmonic cavitation (C_H_) was recorded in real-time throughout the duration of the sonication. Stable harmonic signal was computed for each pulse by taking the root mean square of the maximum signal within a 40 kHz window of the 3rd-6th harmonic. This temporal cavitation data was later used to calculate cavitation dose by calculating the mean bubble cavitation (C_B_) over *n*_*B*_ pulses following bubble injection (CD_B_) (Eq. 1), and the mean control cavitation (C_C_) after *n*_*C*_ control pulses prior to bubble injection (CD_C_) (Eq. 2), then taking the common logarithm of their quotient (Eq. 3) to report a final cavitation dose in dB.

1 $$CD_{B} = \frac{{\sum {C_{B} } }}{{n_{B} }}$$

2 $$CD_{C} = \frac{{\sum {C_{C} } }}{{n_{C} }}$$

3 $$CD = \log_{10} \left( {\frac{{CD_{B} }}{{CD_{C} }}} \right)$$

#### BBBO quantification

The BBBO volume of the first target spot in each mouse was calculated using a semi-automated, threshold-based image segmentation script in Matlab. A z-stack of axial T1-weighted, contrast-enhanced MRI images was analyzed. The region of signal enhancement in each slice, corresponding to Gd diffusion in the BBBO area, was segmented out from the background tissue to create a binary mask. From these segmented images the area of signal enhancement, interpreted as the area of BBBO for a particular slice, was calculated and the masks were interpolated between slices to construct and calculate the three-dimensional BBBO volume.

#### *K*^*trans*^* coefficient & K*^*trans*^* maps*

For each subject, a time-series, z-stack of axial images was fitted to a general kinetic model of diffusion to calculate the K^trans^ permeability coefficient for every pixel of each slice in the 3D stack of the brain. From this K^trans^ map of each axial section in the z-stack, a constant-area ROI encompassing the BBBO region was selected out. These 2D ROIs were then compiled to generate a 3D ROI volume of K^trans^ maps for each animal. The K^trans^ maps were then normalized by the mean background K^trans^ value for the applicable cohort and the mean and maximum K^trans^ values were calculated from the 3D ROI volumes of each animal. The K^trans^ coefficients assigned to each pixel within the ROI volume were then binned into five K^trans^ value ranges based on the global range of values across all six groups. The fraction of the ROI containing K^trans^ values from each bin was averaged across each cohort and plotted for comparison. To generate representative heat maps from K^trans^ maps, the middle slice from each animal’s ROI volume was selected and averaged across the group. The mean middle slice is shown as a 2D representation of the K^trans^ map in the untargeted, background region (Fig. S1C) as well as the target, BBBO region (Fig. 5D).

#### Statistics

Statistical analysis was performed in GraphPad Prism (Version 9.2.0 for macOS, GraphPad Software, San Diego, California USA). Differences between mean stable cavitation dose and mean BBBO volumes were compared by one-way ANOVA with multiple comparisons. Linear regression analysis was performed on BBBC plots, and significant differences between y-intercepts were determined by ANCOVA. One-way ANOVA tests with multiple comparisons were used to compare the average fitted BBBO volumes, the number of days required for BBBC, the ratio of days required for closing to fitted BBBO volume, K^trans^ mean and maximum values, as well as the fraction of the ROI made up of K^trans^ values in each bin. One-way ANOVA tests with multiple comparisons were also used to test for variability in the K^trans^ mean and maximum statistics by age in Fig. S2A, C, and unpaired t tests were used to test for variability between genotypes for the mean and maximum K^trans^ values in Fig. S2B, D. Finally, one-way ANOVA tests with multiple comparisons were used to test for significant differences between 3xTg cohorts in AD-associated protein levels. Significant values are defined as follows: *:*P* ≤ 0.05, **:*P* ≤ 0.01, ***: *P* ≤ 0.001 ****: *P* ≤ 0.0001. All values presented are the mean ± standard deviation unless otherwise stated.

### Protein and plaque quantification

#### Animal sacrifice

Once the BBB was closed, each animal was sacrificed by heavy induction of anesthesia at 5% isoflurane and transcardiac perfusion. The hippocampus was dissected out, rinsed in 1 × PBS, and flash frozen on dry ice. Tissue was stored at − 80 °C until protein quantification.

#### Tissue lysis

Hippocampal tissue was weighed and combined with T-PER solution (1:1000 Halt Protease and Phosphatase Inhibitor Cocktail (ThermoFisher Scientific, Cat#78,446), in T-PER Protein Extraction Reagent (ThermoFisher Scientific, Cat#78,510)) at a concentration of 0.1 mg/μL. The tissue was then homogenized on wet ice, and centrifuged for 30 min at 15,000G at 4 °C. The supernatant, containing soluble protein, was eluted, strained through a 70-μm filter and stored at − 80 °C.

#### Total protein quantification

The concentration of total protein in each sample was quantified using the Pierce™ BCA Protein Assay Kit (ThermoFisher Scientific, Cat#2325). The total protein concentration per sample was used for normalization in subsequent protein quantification.

#### Alzheimer’s disease protein quantification

The amount of Amyloid Beta 40 (Aβ40), Amyloid Beta 42 (Aβ42), phosphorylated tau (pT181) and Total Human Tau was quantified according to manufacturer’s protocol using the bead-based Human ProcartaPlex™ kits from Thermo Fisher Scientific (Cat#EPX010-12,350–901, Cat#EPX010-12,351–901, Cat#EXP010-12,349–901, Cat#EXP010-12,348–901). A standard serial dilution and samples were prepared in duplicates to be read on Luminex 200™ Instrument System (Luminex Corporation, Austin TX). The concentration of each AD analyte was normalized by the total protein concentration for each mouse, and the normalized values were used for statistical analysis.

## Supplementary Information

Supplementary Information.

## Data Availability

The data collected for this study can be made available upon reasonable request to corresponding author Elisa E. Konofagou.
