# Supplementary material for: Natural aging and Alzheimer’s disease pathology increase susceptibility to focused ultrasound-induced blood–brain barrier opening
Source: Sci Rep. 2023 Apr 25;13:6757. doi: 10.1038/s41598-023-30466-6 (PMC10130033; doi:10.1038/s41598-023-30466-6)
Supplement: Supplementary file 1 — Supplementary Information. [file 41598_2023_30466_MOESM1_ESM.docx]

**Supplemental Materials:**

**Figure S1: Control region mean and maximum K^trans^ values do not differ significantly between age and genotype groups.** (A) The mean K^trans^ value within a 3D ROI volume in the cortex (outside of the targeted BBBO region) for each genotype and age group are shown. (B) Maximum K^trans^ values across the 3D ROI volume for each age and genotype group. No statistically significant difference is detected by one-way ANOVA with multiple comparisons for A-B. (C) The average 2D K^trans^ map from the middle slice of a control region ROI volume is shown for each cohort. Color bar scale is determined by the range of K^trans^ values from the BBBO focal region.

**Figure S2: K^trans^ mean and maximum values increase with age and AD progression.** The mean K^trans^ values for pooled, age-matched (A) and genotype-matched (B) cohorts are shown with the standard deviation of each group. The average maximum K^trans^ values for pooled age-matched (C) and genotype-matched (D) cohorts are shown. Statistically significant differences are determined by unpaired t tests.

**Figure S3: Alzheimer’s Disease-associated proteins increase with age in 3xTg cohorts.** The total protein-normalized concentrations of Aβ40 (A), Aβ42 (B), phosphorylated tau (pT181) (C) and total tau (D) and their standard deviations are shown for each 3xTg cohort. Statistically significant differences are determined by one-way ANOVA with multiple comparisons.

**Figure S1**

**
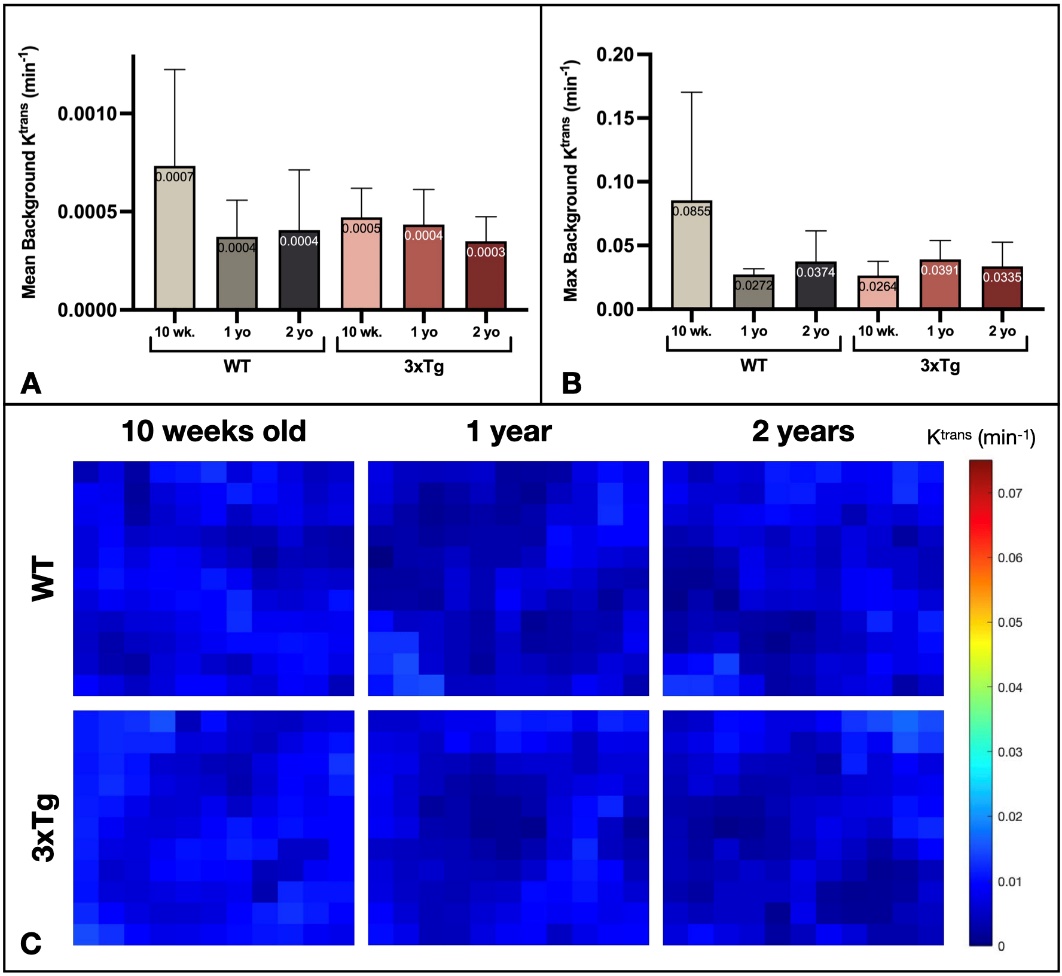
**

**Figure S2**

**
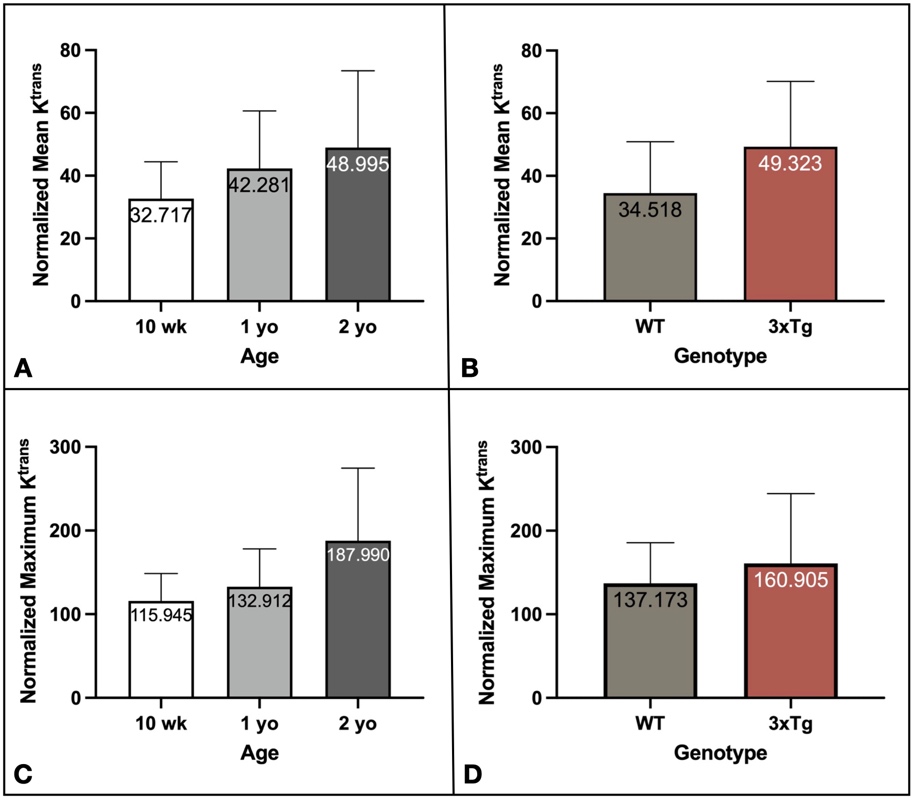
**

**Figure S3**

**
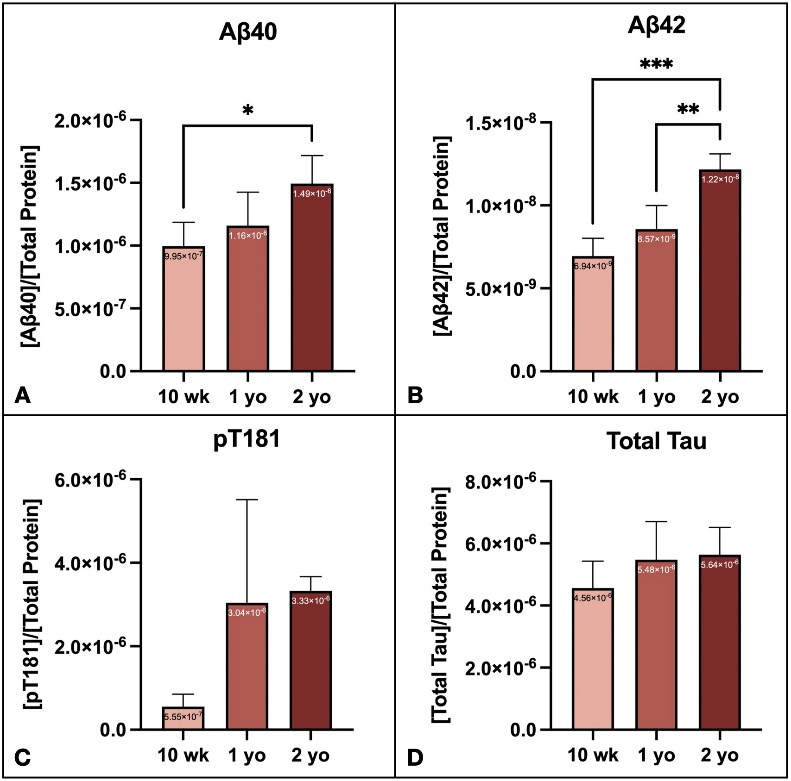
**
